# Axitinib and crizotinib combination therapy inhibits bone loss in a mouse model of castration resistant prostate cancer

**DOI:** 10.1186/1471-2407-14-742

**Published:** 2014-10-02

**Authors:** Jeetendra Eswaraka, Anand Giddabasappa, Guangzhou Han, Kush Lalwani, Koleen Eisele, Zheng Feng, Timothy Affolter, James Christensen, Gang Li

**Affiliations:** Global Science and Technology (WCM), Pfizer Global Research and Development, 10724 Science Center Dr, San Diego, CA 92121 USA; Oncology Research Unit, Pfizer Global Research and Development, 10724 Science Center Dr, San Diego, CA 92121 USA; Drug Safety Research and Development Pfizer Inc, 10724 Science Center Drive, San Diego, CA 92121 USA

**Keywords:** CRPC, Bone metastasis, μCT imaging, X-ray, BLI, Axitinib, VEGFR crizotinib, c-MET

## Abstract

**Background:**

Castration resistant prostate cancer (CRPC) is a leading cause of cancer-related deaths in men. The primary cause of mortality and morbidity in patients is bone metastases and remodeling resulting in osteoblastic and osteolytic lesions. Recently, cabozantinib, a multi-kinase inhibitor (VEGFR2 and c-MET inhibitor), was shown to have efficacy on bone lesions in patients. In this study we tested multi-kinase inhibitors: axitinib (VEGFR inhibitor) and crizotinib (c-MET inhibitor) in a combination trial in mice models.

**Methods:**

VCaP-Luc cells were grown as subcutaneous implants in intact and castrated NOD-SCID-gamma (NSG) mice to confirm the androgen dependency. For bone metastasis model two cohorts of NSG mice (castrated and intact) received orthotopic injection of VCaP-Luc cells into the bone marrow cavity of left tibia. Mice were monitored weekly for tumor growth using bioluminescence imaging. Animals were randomized into 4 groups based on the tumor bioluminescence signal: vehicle, crizotinib alone, axitinib alone, crizotinib and axitinib in combination. Animals were imaged weekly by *in vivo* 2-D X-ray imaging to monitor bone remodeling. At the end of the study animals were euthanized and both tibias were extracted for *ex vivo* high-resolution 3-D micro-computed tomography (μCT) imaging.

**Results:**

Subcutaneous model showed that androgen stimulation may be helpful but not essential for the growth of VCaP-Luc cells. VCaP-Luc cells grown intra-tibially in intact animals caused extensive remodeling of bone with mixed osteoblastic (bone formation) and osteolytic (bone matrix dissolution) lesions. The osteoblastic lesions were predominant and at times extended beyond the tibial shaft into the surrounding tissue. In contrast, only osteolytic lesions were prominent throughout the study in castrated animals. Treatment with crizotinib alone reduced the osteolytic lesions in castrated animals. Axitinib alone reduced the osteoblastic lesions in the intact animals. Combination therapy with axitinib and crizotinib remarkably inhibited the tibial remodeling by VCaP-Luc cells which resulted in a significant reduction of both osteoblastic and osteolytic lesions.

**Conclusion:**

Our data show that combined inhibition of c-MET and VEGFR can be beneficial for treatment of metastatic bone disease in CRPC and that the drugs act on two different stages of the disease.

**Electronic supplementary material:**

The online version of this article (doi:10.1186/1471-2407-14-742) contains supplementary material, which is available to authorized users.

## Background

Prostate cancer (PC) is the second leading cause of new cancer cases in men worldwide. According to the Centers for Disease Control and Prevention (CDC), PC is the most commonly diagnosed malignancy and the second leading cause of cancer related deaths among men in the USA [1]. Due to advances in screening and prevention, most PC cases are detected at early stages. Although localized PC can be treated with one or a combination of therapeutic modalities [2], many patients nonetheless go on to develop metastatic disease, especially in the local lymph nodes and bones. Currently androgen deprivation therapy (ADT) represents the primary treatment for metastatic PC. Unfortunately, after an initial benefit from androgen deprivation, PC often progresses after 12–48 months resulting in a more aggressive castration resistant prostate cancer (CRPC) form of the disease [3].

The primary cause of mortality and morbidity in CRPC patients is bone metastases, with local disruption of normal bone remodeling secondary to an increase in osteoblastic and osteolytic lesions. Metastatic bone lesions eventually lead to bone weakening which can result in skeletal fractures, spinal cord compression, intractable bone pain, cachexia and death [4]. The standard of care (SOC) therapy for patients at this stage of disease is chemotherapy (Docetaxel) with or without prednisone [5]. Chemotherapy improves survival but has no effect on the metastatic bone lesions. In addition, osteoblastic or mixed (osteoblastic and osteolytic) metastatic bone lesions associated with PC are poorly responsive to osteoclast inhibitors [6]. Therefore, a treatment that can reduce primary prostatic tumors and metastatic bone lesions could provide a significant improvement in the quality of life for patients with advanced disease [7].

Recently, a multi-targeted tyrosine kinase inhibitor cabozantinib (XL-184) [8] was shown to reduce bone lesions, improve pain management and offer some survival benefit in CRPC patients. It was proposed that the observed effect was due to dual inhibition of vascular endothelial growth factor receptor 2 (VEGFR2) and hepatocyte growth factor (HGF) receptor c-MET [9], however the exact mechanism of action of cabozantinib remains to be further understood. It is known that angiogenesis plays an important role in PC progression and metastasis [10, 11]. VEGFR expression has been associated with worse prognosis in several cancers, including prostate cancer [12]. In addition to its effect on endothelial cells, VEGFR activation also stimulates osteoblast migration [13] and differentiation [14], suggesting that VEGF/VEGFR pathway may contribute to bone lesions in metastatic PC [15, 16]. Under normal conditions, c-MET is an important mediator of organogenesis, tissue regeneration and angiogenesis. In the normal prostate epithelium, c-MET is specifically expressed in basal and atrophic luminal cells [17]. In PC, an increase in c-MET expression was observed [18, 19] and appeared to be associated with progression of bone metastasis [20, 21]. Moreover, an inverse relationship between androgen level and c-MET expression has been observed in prostate cancer cell lines [18], suggesting that androgen deprivation, the current approach to metastatic PC may negatively contribute to disease response by increasing the expression of c-MET [22].

To gain additional insight into the potential clinical relevance of targeting both VEGFR2 and c-MET in men with PC bone metastases, we tested the effect of axitinib (VEGFR inhibitor) [23] and crizotinib (c-MET inhibitor) [24], either as single agents or in combination, in an orthotopic bone metastasis model of PC in both intact and castrated NSG mice [25]. In intact mice, VCaP-Luc cell infiltration into bone caused extensive remodeling of bone with mixed lesions (osteoblastic and osteolytic), whereas in castrated animals osteolytic lesions were more prominent indicating a potential role of androgen in the bone phenotype of the disease. Treatment with crizotinib alone reduced the osteolytic lesions seen in castrated animals. Axitinib alone reduced the osteoblastic lesions in the intact animals. Combination therapy with axitinib and crizotinib had a remarkable effect in inhibiting the tibial remodeling with a reduction of both osteoblastic and osteolytic lesions. These results show that combined inhibition of c-MET and VEGFR2 can be beneficial for the treatment of metastatic bone disease in a CRPC model and that the drugs act on two different stages of the disease.

## Methods

### Compounds

The following compounds were generated at Pfizer: axitinib (AG013736; trade name Inlyta®) [23] and crizotinib (PF-02341066; trade name Xalkori®) [24].

### Cell line and animals

Human prostate cancer line VCaP was obtained from American Type Culture Collection (catalog #CRL-2876; ATCC, Manasas, VA) and maintained in Dulbecco’s Modified Eagle’s Medium (DMEM) supplemented with 10% fetal bovine serum (FBS). VCaP-Luc cell line was made by transfection with vector pLPCX: Luc-SH and selected with puromycin. Male NOD.Cg-*Prkdc*^*scid*^*Il2rg*^*tm1Wjl*^/SzJ (NSG) mice which were 6–8 week old was purchased from Jackson Laboratory (Bar Harbor, ME) and housed in barrier rooms under pathogen-free conditions. All animal experiments were carried out in compliance with Pfizer’s Institutional Animal Care and Use Committee (IACUC) guidelines and in accordance with the “Guide for the Care and Use of Laboratory Animals” by the Institute of Laboratory Animal Research Commission on Life Sciences (ILARCLS, National Research Council, Washington D.C.). All animal handling and surgical procedures were performed using protocols approved by Pfizer’s IACUC. For castration, animals were anesthetized using Ketamine/Xylazine via intraperitoneal route and castration was performed by a scrotal incision procedure. Animals were allowed to recover from surgery for 2 weeks before being enrolled in experiments.

### Subcutaneous tumor model

VCaP-Luc cells (1 × 10^6^ cells/animal) were implanted subcutaneously into the flank of ~10 week old male NSG mice. Ten of the NSG mice were castrated before cell implantation (labeled as “castrated”), ten were castrated 5 weeks after cell implantation (labeled as “intact-castrated”) and ten received sham surgery (labelled as “intact”). Tumor volume was calculated based on weekly caliper measurement using the formula: volume = (width)^2^ × length /2.

### Intra-tibial tumor model

Orthotopic bone tumor model was performed based on Park et al. [26] and Corey et al. [27] with minor modifications. Briefly, ~10 week old male NSG mice with or without castration were anesthetized by administering a mixture of ketamine and xylazine via intraperitoneal route. A small hole was drilled proximal to the tibial tuberosity with a 19-gauge needle. After penetration of the cortical bone, an intraosseous injection of 30ul sterile PBS containing 1 million VCaP-Luc cells in suspension was administered. Tumor growth was monitored by plasma PSA, bioluminescence imaging (BLI by IVIS-200) and X-ray (by Faxitron). Drug treatment was initiated 4 weeks after tumor implantation. Tumor-bearing mice were randomized based on BLI measurements and enrolled into 4 treatment groups (n = 10 mice/group): vehicle, crizotinib (50 mg/kg, oral QD) only, axitinib (30 mg/kg, oral BID) only and crizotinib (50 mg/kg, oral QD) + axitinib (30 mg/kg, oral BID). At the end of the study, tibias of both the control and tumor bearing limb from all animals was collected for μCT analysis. The bones were immersed in 10% neutral buffered formalin (NBF) for 3 days for fixation. The tissues were then removed from NBF and wrapped in saline soaked gauze for storage at -80°C until μCT analysis.

### Immunohistochemistry

Tibias from intact or castrated mice bearing VCaP-Luc tumor were collected, fixed in 10% NBF, decalcified, paraffin embedded and sectioned at a thickness of 5 μm. Slides were baked, loaded onto Leica Bond III automated IHC/ISH strainer (Leica Microsystems, Inc., Bannockburn, IL), and deparaffinized with BOND dewaxing solution (Leica Microsystems). Antigen retrieval was achieved by exposing the tissue slides to Epitope Retrieval Solution 2 (Leica Microsystem) for 30 minutes, and then quenching endogenous peroxidase by incubating with 3% hydrogen peroxide. After multiple washing steps, slides were incubated with primary antibody (rabbit anti-human c-MET monoclonal antibody, clone SP44) (Spring Bio/Ventana/Roche, Pleasanton, CA) or rabbit IgG as negative control (Cell Signaling Technology, Inc., Danvers) for 60 minutes and detected by using Bond Polymer Refine Detection Kits (Leica Microsystems), followed by counterstaining with hematoxylin. Slides were dehydrated, cleared, and mounted with cover slips. All the images were visualized using Nikon ECLIPSE E-400 Microscope (Nikon Microscope) and captured through a SPOT Insight Color Mosaic 11.2.1 camera using SPOT Software, V4.7.

### Western blotting

Subcutaneous tumor samples were collected and frozen immediately in liquid nitrogen when they reached 800 mm^3^. To prepare lysate, snap-frozen tumor fragments were lysed in FastPrep Lysing Matrix A tubes according to manufacturer’s recommendations (MP Biomedicals, Santa Ana, CA). Following protein measurement by BCA (Thermo Scientific, Waltham, MA), 20 μg of each lysate was electrophoresed, transferred to nitrocellulose membrane using the iBlot Dry Blotting System (Invitrogen, San Diego CA). The anti-androgen receptor antibody PG-21 (EMD Millipore, Temecula, CA) was used as primary antibody for detecting AR. Anti-β-actin antibody AC15 (Sigma-Aldrich, St. Louis, MO) was used as control.

### ELISA measurement of plasma PSA

Plasma was obtained every 2 weeks for measurement of PSA levels using Quantikine human PSA immunoassay kit (R&D Systems, Minneapolis, MA) as per manufacturer’s instructions.

### Bone imaging and analysis

Mouse tibias were initially imaged by X-ray (Faxitron MX20; Faxitron Bioptics, LLC; Tuscon, Arizona) once a week throughout the study using the following parameters: 20kv and 5 s exposure time. At the end of the study tibias from both legs were collected and imaged *ex vivo* by μCT (vivaCT 75; Scanco Medical AG; Basserdorf, Switzerland) in high resolution using the following parameters: 70kVP, 114 μA, 300 ms integration time, 20.5-μm voxel size. The scanning protocol was programmed to acquire images via a rotating gantry, resulting in a total of 2,000 projections per scan. The projections were reconstructed with a matrix of 2048 × 2048 using the software provided by Scanco Medical. A 615-μm-thick cross-section of the cancellous bone at the proximal tibial epiphysis, including the primary and secondary spongiosa, was taken ~2.0 mm proximal to the growth plate. An optimized input threshold of 175 was selected over the software default threshold of 220 based on a visual assessment of the quality of segmentation. Bone structural parameters such as connectivity density (Conn.D), structure model index (SMI), bone mineral density (BMD), bone volume fraction (BV/TV), and trabecular number, thickness, and separation (Tb.N, Tb.Th, and Tb.Sp, respectively) were ascertained using Scanco’s μCT Evaluation Program V6.5-1. Images were later converted to 2D binary digital imaging communication (DICOM) files and imported into 64-bit OsiriX (Opensource software) for image reconstruction.

### Statistical analysis

Data are expressed as mean ± SEM unless indicated otherwise. Statistical significance was determined by analysis of variance (ANOVA) using Dunnett’s multiple-comparison post test with GraphPad Prism® software unless otherwise noted.

## Results

### Characterization of VCaP-Luc as a subcutaneous model

**Figure 1:**
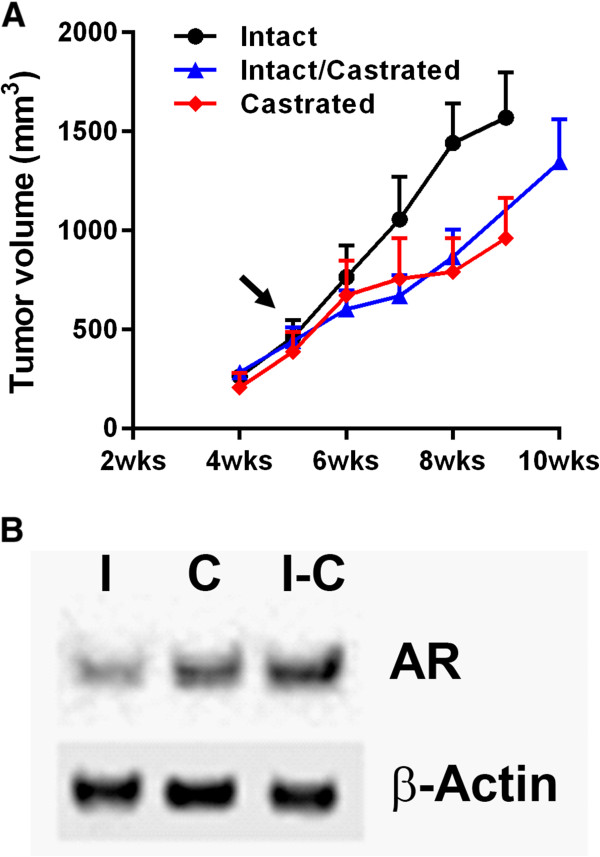
**Characterization of subcutaneous xenograft model of VCaP-Luc. (A)** Tumor growth curves of VCaP-Luc cells subcutaneously implanted in intact (sham surgery), castrated (castrated before tumor implantation) and intact-castrated mice (castrated 5 weeks after tumor implantation, as indicated by the arrow). Error bar represents SEM of n = 10 mice/group. **(B)** Western blot of androgen receptor expression in VCaP-Luc subcutaneous tumors grown in intact (I), castrated (C) and intact-castrated (I-C) mice. Tumors were harvested at approximately 800 mm^3^. Representative image from 3 tumor samples.

To facilitate longitudinal measurement of the orthotopically grown tumors, we generated bioluminescence capable cell line VCaP (VCaP-Luc) by transfection with a luciferase-encoding vector. VCaP-Luc was first grown as a subcutaneous model in NSG mice (intact and castrated) to evaluate its androgen dependency and growth characteristics. Palpable tumors were seen about 4 weeks after implantation (Figure 1A). Tumors grew at a slightly faster rate in the intact animals suggesting that the cell line is responsive to androgen stimulation. Half of these intact animals were castrated at 5 weeks post implantation to observe the effect of androgen deprivation. Following castration the tumors showed a delayed growth rate but were still able to grow to size of 1500 mm^3^ by 10 weeks post-implantation (Figure 1A). A similar effect was seen with animals that had been castrated prior to tumor implantation (Figure 1A). At the end of the study tumors were evaluated for androgen receptor (AR) expression using western blot analysis. Data (Figure 1B) show that the tumors in all three groups have detectable levels of AR with a slight increase in expression in the castrated animals. These results indicate that the VCaP-Luc cell lines maintain the parental characteristics post-transfection with luciferase gene, and that androgen stimulation is helpful but not essential for growth of the cell line.

### Intra-tibial model characterization

**Figure 2:**
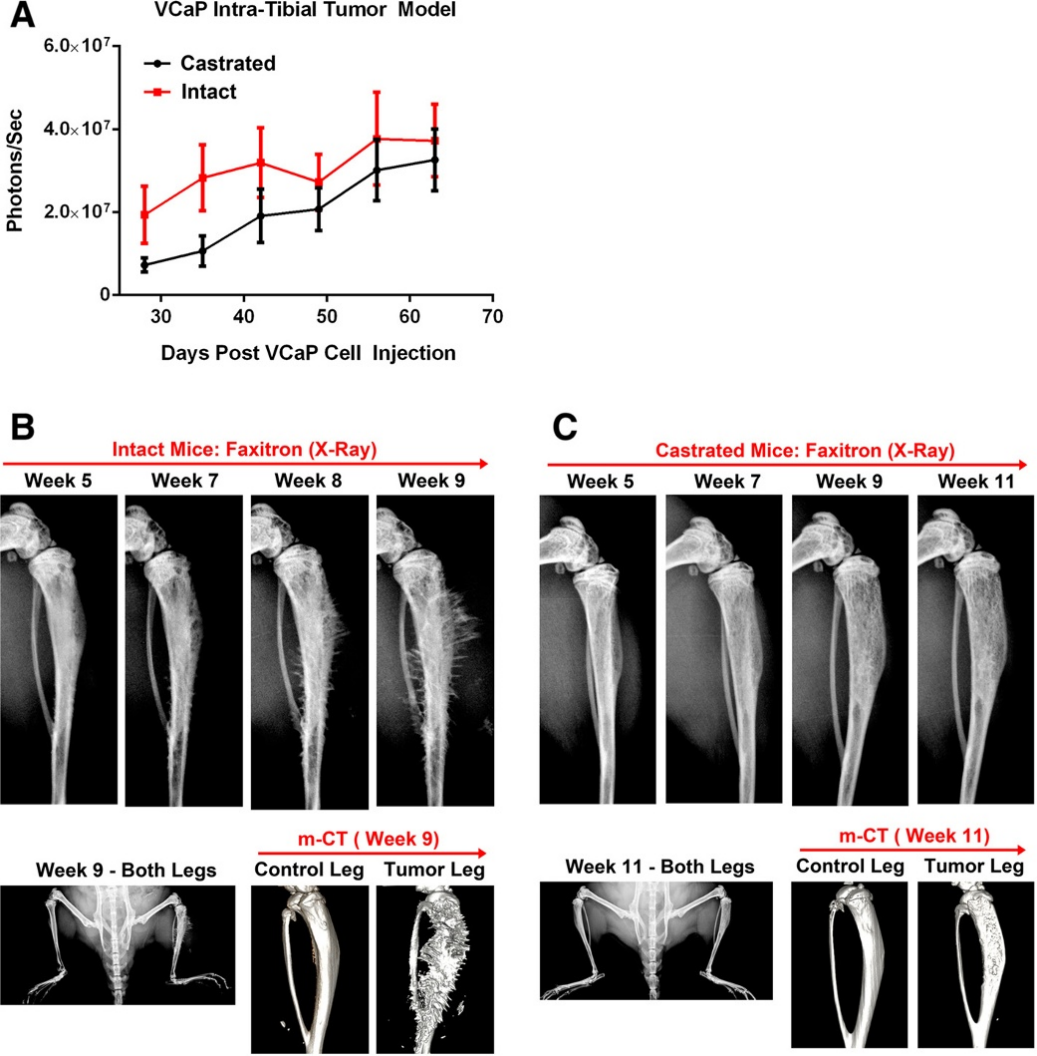
**Characterization of intra-tibial bone metastasis model of VCaP-Luc. (A)** Measurement of intra-tibial tumor growth in intact (sham surgery) and castrated (castrated before intra-tibial injection of tumor cells) NSG mice by bioluminescence imaging once a week. Error bars represent SEM of n = 10 mice/group. **(B)** Faxitron and μCT imaging of intra-tibial PC model in intact mice. Faxitron imaging (*in vivo*) was performed at indicated time intervals during the study. The inset of Faxitron imaging shows control leg (no intra-tibial tumors) and tumor leg (received VCaP cell intra-tibially) at week 9. *Ex vivo* μCT imaging was performed and reconstructed at the termination of the study (week 9). Control leg and tumor legs after 3-D reconstruction is shown. **(C)** Faxitron and μCT imaging of intra-tibial PC model in Castrated mice. Faxitron imaging (*in vivo*) was performed at indicated time intervals during the study. The inset of Faxitron imaging shows control leg (no intra-tibial tumors) and tumor leg (received VCaP-Luc cells intra-tibially) at week 9. *Ex vivo* μCT imaging was performed and reconstructed at the termination of the study (week 9). Control leg and tumor legs after 3-D reconstruction is shown.

Following intra-tibial injection of VCaP-Luc cells to NSG mice (intact and castrated) as described in Materials and Methods section, tumor growth was monitored using bioluminescent imaging (BLI) and Faxitron imaging (X-ray). The results showed that tumor growth rates in this model were different between the castrated and intact animals. Tumor growth in intact mice was detectable 4 weeks post-implantation using BLI with an average luminescence intensity of 2 × 10^7^ photons/second. Tumors continued to progress and reached 3 × 10^7^ photons/second by 9 weeks post-implantation (Figure 2A). Faxitron imaging of these tibias showed that during the observation period, there was an initial loss of bone density between 5–7 weeks (Figure 2B). However, by about 8 weeks bone proliferation was seen by increased bone density and formation of bone spicules radiating from the bone shaft. By about 9 weeks some of these proliferative bone lesions were seen to extend out into the surrounding soft tissue space. At this stage, the animals reached the end point for lameness and had to be terminated. In castrated mice, the tumors grew more slowly and BLI was detectable (1 × 10^7^ photons/second) between 5–7 weeks post-implantation in all animals. The tumors continued to progress and reached 3 × 10^7^ photons/second by 10–11 weeks. Faxitron imaging of the implanted tumors in these animals showed a predominantly osteolytic phenotype with loss of bone density throughout the 11 weeks of monitoring. Animals had to be euthanatized at this point due to lameness endpoint (Figure 2C). Histopathologic section of the tumor-laden tibia revealed regionally extensive invasion and effacement of normal bone architecture extending from within the marrow cavity into the adjacent epiphysis, metaphysis, diaphysis, and growth plate. Areas of osteolysis admixed with occasional osteoclasts and areas of attempted repair with plump osteoblasts are interspersed throughout regions of cancellous and cortical bone (Additional file 1: Figure S1). C-Met expression in the tumor cells was seen in both intact and castrated tibias. Interestingly, the c-Met staining was both diffusely cytoplasmic and membranous in the intact mice tibias, while in castrated mice the staining was predominantly membranous (Additional file 2: Figure S2). This differential expression and localization could be indicative of the different cell phenotypes seen in the bone of these mice.

### Effect of targeting VEGFR and c-MET in VCaP-Luc intra-tibial tumor model

**Figure 3:**
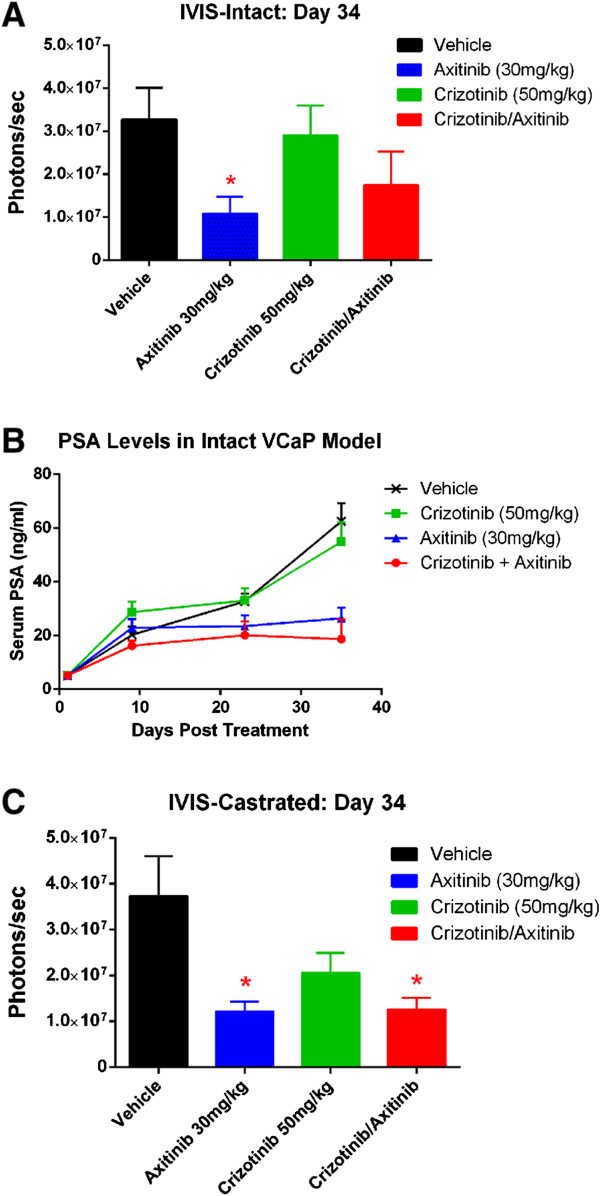
**Efficacy of axitinib and crizotinib in intact and castrated tumor bearing mice by bioluminescence imaging. (A)** Bioluminescence measurement of treatment groups on day 34 in intra-tibial VCaP tumor model in intact mice. **(B)** PSA levels during the course of treatment in intact mice bearing intra-tibial VCaP-Luc tumor. **(C)** Bioluminescence measurement of treatment groups on day 34 in intra-tibial VCaP tumor model in castrated mice. PSA levels in all groups of castrated mice were below level of detection (data not shown). *p < 0.01, compared to vehicle group. Error bar represents SEM of n = 10mice/group.

We next tested the efficacy of kinase inhibitors targeting VEGFR (axitinib) and c-MET (crizotinib) in this model. In addition to BLI and X-ray imaging modalities, we also used *ex vivo* μCT at the end of the study to characterize bone phenotype. Tumor evaluation using BLI imaging showed that treatment of intact tumor-bearing animals with axitinib alone or axitinib and crizotinib in combination led to statistically significant reduction in tumor progression compared with vehicle treated animals by day 34 (Figure 3A). This reduction in tumor size correlated to a lower plasma PSA levels between Day 10 and the end of the study in these groups compared to vehicle treated animals and animals receiving crizotinib alone (Figure 3B). Animals treated with crizotinib alone had no statistically significant reduction in tumor size (Figure 3A) and showed no reduction in plasma PSA levels (Figure 3B) indicating that targeting c-MET alone is not sufficient. In the castrated group, axitinib alone or axitinib in combination with crizotinib resulted in a statistically significant reduction in tumor burden by day 34 (Figure 3C). Though there was a visible reduction in tumor burden in crizotinib alone group, it was not statistically significant (Figure 3C). PSA levels in the castrated mice were below the threshold detection level of 10-20 ng/ml in all groups (data not shown) and thus suggest PSA may not predict the tumor burden.

**Figure 4:**
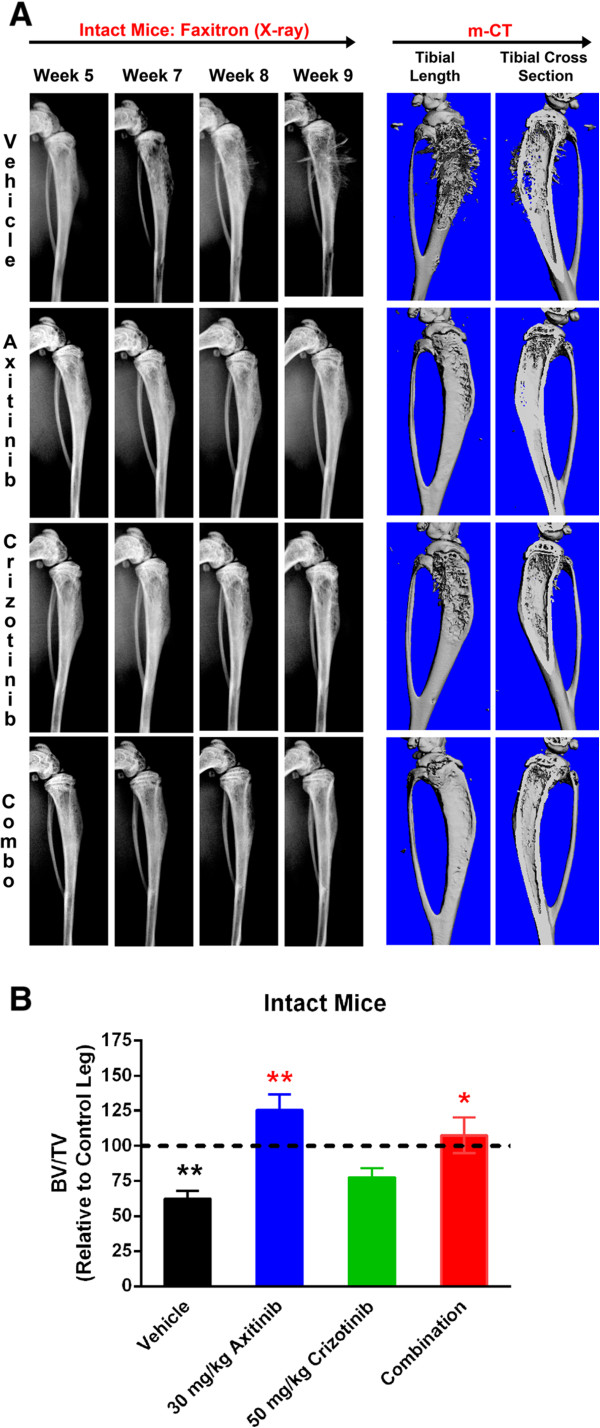
**Efficacy of axitinib and crizotinib in intact tumor bearing mice by** ***in vivo*** **Faxitron X-ray and** ***ex vivo*** **μCT imaging. (A)** Faxitron and μCT imaging in intact mice model. Faxitron images were taken at indicated time intervals during the study, whereas μCT images were taken and reconstructed at the termination of the study (week 9). μCT images show the 3-D of the tibial length and a tibial cross-section. **(B)** Quantitation of Intra-tibial tumor model in Intact mice: BV/TV ratios were determined by Scanco software after μCT imaging. ** (black) is vehicle vs control leg; * (red) treatment vs vehicle. *p < 0.01; **p < 0.001. Error bars represent SEM of n = 10 mice/group.

**Figure 5:**
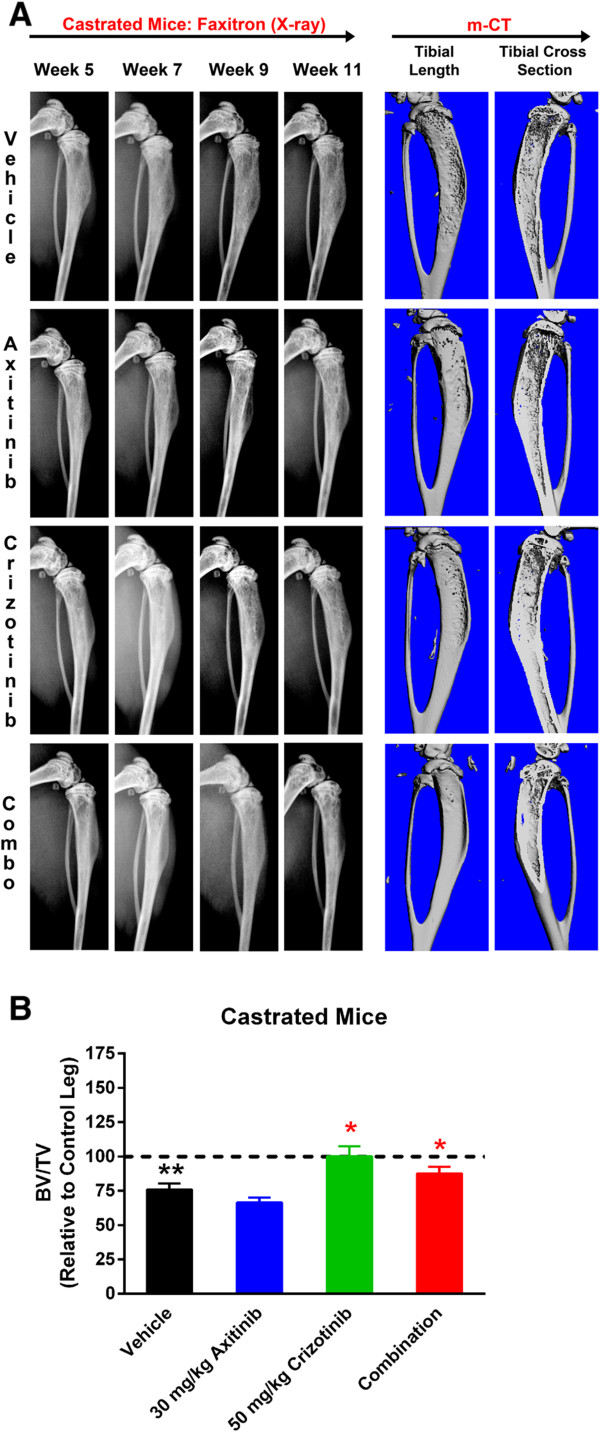
**Efficacy of axitinib and crizotinib in castrated tumor bearing mice by** ***in vivo*** **Faxitron X-ray and** ***ex vivo*** **μCT imaging.** **(A)** Faxitron and μCT imaging in castrated mice model. Faxitron images were taken at indicated time intervals during the study, whereas μCT images were taken and reconstructed at the termination of the study (week 9). μCT images show the 3-D of the tibial length and a tibial cross-section. **(B)** Quantitation of Intra-tibial tumor model in Castrated mice: BV/TV ratios were determined by Scanco software after μCT imaging. ** (black) is vehicle vs control leg; * (red) treatment vs vehicle. *p < 0.01; **p < 0.001. Error bars represent SEM of n = 10 mice/group.

Image analysis (assessed by Faxitron and μCT imaging) of the tumor bearing tibias showed that intact animals in vehicle-treated group developed mixed osteolytic and osteoblastic tumor-associated lesions (Figure 4A). Despite the presence of extensive proliferative bone lesions on the X-ray images, bone density was poor compared to that of the normal bone (control right leg) and the bone volume (ratio of bone volume to trabecular volume (BV/TV)) was approximately 35% less than that of the normal bone (Figure 4B). In the axitinib treated group, Faxitron imaging from 5–9 weeks showed that there was very little loss of bone tissue contrast and no significant bony proliferation. *Ex vivo* μCT analysis of the tibias on the other hand showed a mild increase in the bony proliferation exhibited as scalloping of the bone diaphysis (Figure 4A). In the crizotinib alone treated group, proliferative osteoblastic lesions were very evident and similar to that of the vehicle treated groups. Additionally the areas of osteolysis in the bone were less than that observed in the vehicle treated animals (Figure 4A). In the animals treated with the combination of axitinib and crizotinib the tumor bearing bones had very little evidence of osteoblastic or osteolytic bone remodeling (Figure 4A) indicating that this combination therapy was very effective in the prevention of bone remodeling induced by the tumor in intact mice. Additionally, the phenotype and BV/TV ratios were similar in axitinib alone or combo therapy groups. In the vehicle-treated castrated cohort, Faxitron images showed a gradual reduction in bone density from week 5 to 11 (Figure 5A). *Ex vivo* μCT of the bones confirmed the osteolytic lesions in vehicle-treated mice (Figure 5A). The bone density was poor compared to that of the normal bone (control right leg) and the BV/TV ratio was approximately 25% less than that of the normal bone (Figure 5B). Treatment with axitinib alone did not have any effect on the loss of radiodensity on Faxitron, osteolytic lesions or BV/TV ratio by the μCT (Figure 5A and 5B) imaging. In the crizotinib alone or crizotinib and axitinib combination groups the loss of bone density on Faxitron and μCT was very minimal, indicating that these treatments prevented the bone loss initiated by the tumor proliferation (Figure 5B).

## Discussion

Prostate Cancer (PC) represents a major health issue in men with an estimated 240,000 new cases and almost 30,000 deaths annually in the United States [28]. Although surgery and radiation therapy has cured many patients with PC, more than one third of the patients will eventually progress and develop advanced disease, for which ADT is the standard of care. Unfortunately, most patients who undergo chemical or surgical castration will eventually progress and develop CRPC [29, 30]. There have been numerous studies attempting to understand the mechanisms and processes for the development of CRPC, some of which include ligand-independent AR activation, AR mutations, amplification and selection of pre-existing androgen independent cells that are resistant to apoptosis [31, 32].

Bone is the most common site of metastases in men with advanced PC. Historical data shows that the overwhelming majority (more than 80%) of PC patients eventually develop bone disease [6, 7, 33]. Cancer metastases in the bone usually lead to disturbance of bone formation and resorption, resulting in osteolytic and/or osteoblastic lesions [34–36]. It remains largely unclear what factors determine whether a particular bone metastasis is osteolytic, osteoblastic or of mixed phenotype, although some studies suggested that different types of cancer have the propensity to secrete more osteoblastic or osteolytic factors [37]. In this study, we observed different bone phenotypes between castrated and intact host mice intra-tibially implanted with VCaP-Luc cell line. Although this cell line is classified as a castration-resistant prostate cancer line, its growth rate was reduced in castrated mice compared with intact mice, in both subcutaneous and intra-tibial growth conditions. This difference may lead to difference in growth characteristics of the cells and in the type of paracrine factors that affect the activation of osteoblasts and osteoclasts. Regardless of the phenotype, bone metastases usually lead to compromised bone integrity and strength, and as a result, increased incidence of fractures, spinal cord compression and severe pain [38, 39]. These are not only quality of life issues, but also directly worsen the survival span of the patients. Thus, effective prevention and management of bone metastases is critical for reducing PC related morbidity and mortality. Until recently, there have been limited choices for therapeutic intervention of metastasis-induced bone loss. Although zoledronic acid has been used to prevent bone loss, its long term benefit is not well defined [40]. Newer agents such as Denosumab [41] and cabozantinib [42] have shown clinical benefit in delaying the occurrence of skeletal-related events (SRE) in PC patients.

Crizotinib is a potent and selective inhibitor of c-MET and several related tyrosine kinases. C-MET and its ligand, hepatocyte growth factor (HGF), are involved in cell proliferation, differentiation, motility and survival [43]. Crizotinib exhibited a dose-dependent anti-tumor effects by inhibition of tumor cell proliferation, micro vessel density and angiogenesis [44]. Tumor effects due to in prostate cancer, c-MET expression is elevated in bone metastases compared with lymph node metastases or primary tumors [19], and in tumors in castrated patients compared with non-castrated patients [45]. In addition, c-MET pathway is believed to play a role in proliferation, differentiation and migration of osteoblasts and osteoclasts [46, 47]. The c-MET ligand, HGF can be secreted by multiple cell types including osteoclasts and mesenchymal cells, potentially forming autocrine and paracrine regulation of bone remodeling [46]. Axitinib the other kinase inhibitor used in this study is a potent and selective inhibitor of angiogenesis targeting VEGFR2. Elevated levels of circulating VEGF have been correlated with poor prognosis in PC patients [48]. VEGF and its receptors are shown to be expressed in osteoblasts and osteoclasts [49], exerting multiple functions through autocrine and paracrine activation of the VEGFR pathway. Taking into account the function of c-MET and VEGFR in PC metastasis, we hypothesized that blocking both pathways simultaneously by combining two targeted agents may offer substantial benefit to PC patients. Our data showed that axitinib alone or axitinib and crizotinib in combination reduced the tumor burden in both intact (androgen positive) and castrated (androgen negative) mice models (Figure 3A and 3C). Interestingly only the axitinib and crizotinib combination showed improvements of bone volume (BV/TV ratio) in both models (Figure 4B and 5B). This suggested that combination therapy had clear benefit on tumor burden and bone volume in both intact (androgen positive) and castrated (androgen negative) mice models of PC. The combination can also be achieved by a single agent with dual or multiple specificities as indicated by cabozantinib, a potent inhibitor of receptor tyrosine kinases including MET, VEGFR2, AXL, FLT-3, KIT, and RET [9]. In fact, a phase II adaptive, randomized discontinuation trial of cabozantinib in patients with metastatic CRPC [42] has generated encouraging results showing clear improvements in bone scans in 68% of evaluable patients. On the other hand, co-administration of two separate compounds (such as crizotinib and axitinib) would allow flexibility when the dosage of each compound or pathway needs modification.

## Conclusion

Our results show that co-administration of c-MET (Crizotinib) and VEGFR2 (Axitinib) inhibitors suppressed tumor growth and maintained bone phenotype. Combined inhibition of c-MET and VEGFR can be beneficial for treatment of metastatic bone disease in CRPC and that the drugs act on two different stages of the disease.

## Electronic supplementary material

Additional file 1: Figure S1: H and E staining of the normal tibia and intra-tibial VCaP model of PC. Tumor bearing tibias show infiltration of osteoblasts and osteoclasts into the growth plate, epiphysis and diaphysis. (TIFF 2 MB)

Additional file 2: Figure S2: Differential c-Met immunostaining in tumor bearing intact and castrated tibias. Intact mice tibias showed both diffused cytoplasmic and membranous staining of c-MET, whereas in castrated mice the c-MET staining was primarily at the cell membrane. (TIFF 3 MB)

## Abbreviations

ADT: Androgen deprivation therapy
BLI: Bioluminescence
BV: Bone volume
CRPC: Castration resistant prostate cancer
μCT: Micro-computed tomography
NBF: Normal buffered formalin
PC: Prostate cancer
TV: Trabecular volume
SOC: Standard of care.
