# Supplementary figures and images for: Axitinib and crizotinib combination therapy inhibits bone loss in a mouse model of castration resistant prostate cancer
Source: BMC Cancer. 2014 Oct 2;14:742. doi: 10.1186/1471-2407-14-742 (PMC4190397; doi:10.1186/1471-2407-14-742)

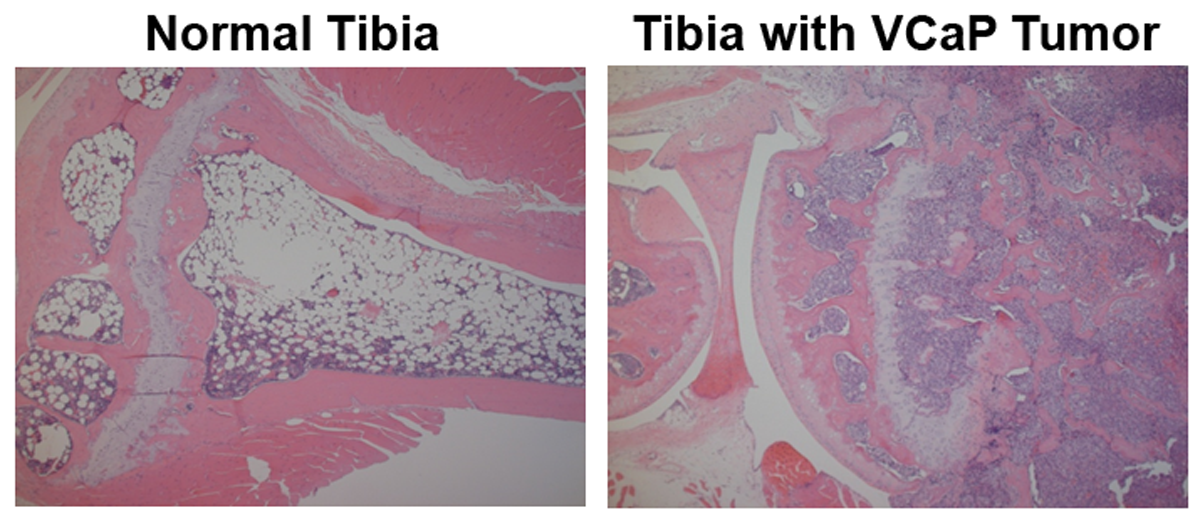

Supplement: Supplementary file 1 — Additional file 1: Figure S1: H and E staining of the normal tibia and intra-tibial VCaP model of PC. Tumor bearing tibias show infiltration of osteoblasts and osteoclasts into the growth plate, epiphysis and diaphysis. (TIFF 2 MB) [file 12885_2014_4915_MOESM1_ESM.tiff]

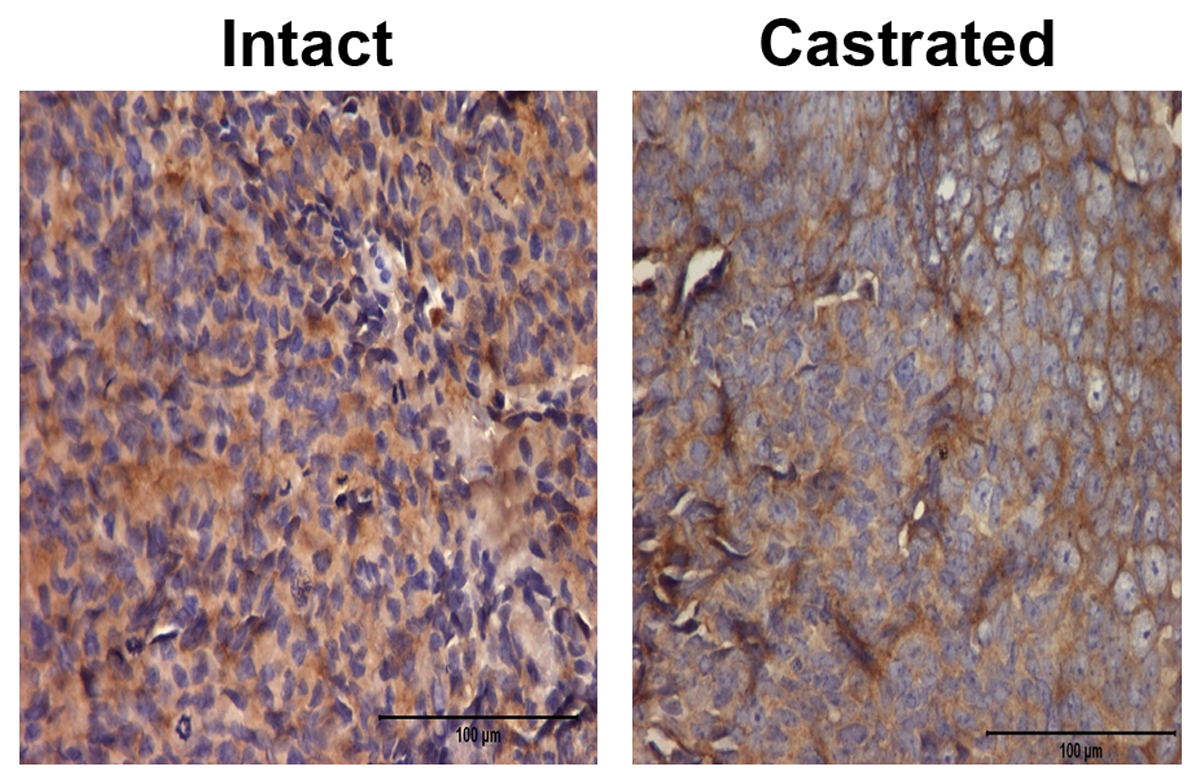

Supplement: Supplementary file 2 — Additional file 2: Figure S2: Differential c-Met immunostaining in tumor bearing intact and castrated tibias. Intact mice tibias showed both diffused cytoplasmic and membranous staining of c-MET, whereas in castrated mice the c-MET staining was primarily at the cell membrane. (TIFF 3 MB) [file 12885_2014_4915_MOESM2_ESM.tiff]
